# Feasibility of CT attenuation values in distinguishing acute ischemic stroke, old cerebral infarction and leukoaraiosis

**DOI:** 10.1186/s12880-024-01340-2

**Published:** 2024-06-26

**Authors:** Yun Peng, Chunyuan Luo, Heng Wang, Ke Sun, Fang Lin, Jingzhi Wang, Yutong Rao, Ruoyun Fan, Lianggeng Gong, Xiaoyu Sun

**Affiliations:** 1 Department of Radiology, The Second Affiliated Hospital, Jiangxi Medical College, Nanchang University, No. 1 Minde Road, Nanchang, Jiangxi 330001 China; 2 Intelligent Medical Imaging of Jiangxi Key Laboratory, Nanchang, 330006 China

**Keywords:** CT, Acute ischemic stroke, Leukoaraiosis, Old cerebral infarction

## Abstract

**Purpose:**

This study aimed to investigate the feasibility of using computed tomography (CT) attenuation values to differentiate hypodense brain lesions, specifically acute ischemic stroke (AIS) from asymmetric leukoaraiosis (LA) and old cerebral infarction (OCI).

**Materials and methods:**

This retrospective study included patients with indeterminate hypodense lesions identified via brain CT scans conducted between June 2019 and June 2021. All lesions were confirmed through head MRI/diffusion-weighted imaging within 48 h after CT. CT attenuation values of hypodense lesions and symmetrical control regions were measured. Additionally, CT attenuation value difference (ΔHU) and ratio (Ratio_HU_) were calculated. One-way analysis of variance (ANOVA) was used to compare age and CT parameters (CT attenuation values, ΔHU and Ratio_HU_) across the groups. Finally, receiver operating characteristic (ROC) analysis was performed to determine the cutoff values for distinguishing hypodense lesions.

**Results:**

A total of 167 lesions from 146 patients were examined. The CT attenuation values for AIS(*n* = 39), LA(*n* = 53), and OCI(*n* = 75) were 18.90 ± 6.40 HU, 17.53 ± 4.67 HU, and 11.90 ± 5.92 HU, respectively. The time interval between symptom onset and CT scans for AIS group was 32.21 ± 26.85 h. ANOVA revealed significant differences among the CT parameters of the hypodense lesion groups (all *P* < 0.001). The AUC of CT values, ΔHU, and Ratio_HU_ for distinguishing AIS from OCI were 0.802, 0.896 and 0.878, respectively (all *P* < 0.001). Meanwhile, the AUC for distinguishing OCI from LA was 0.789, 0.883, and 0.857, respectively (all *P* < 0.001). Nevertheless, none of the parameters could distinguish AIS from LA.

**Conclusion:**

CT attenuation parameters can be utilized to differentiate between AIS and OCI or OCI and LA in indeterminate hypodense lesions on CT images. However, distinguishing AIS from LA remains challenging.

## Introduction

Acute ischemic stroke (AIS) is a type of cerebrovascular disease characterized by high morbidity and disability rates, accounting for > 80% of all strokes [1, 2]. The treatment objective for AIS is to promptly restore blood flow in occluded blood. Therefore, early and accurate diagnosis is crucial [2, 3]. The diagnosis of AIS can be made by excluding cerebral hemorrhage in patients with sudden onset hemiplegia, aphasia, and hemianopsia. However, for patients who do not manifest these severely disabling symptoms and only presenting with dizziness or weakness, a diagnosis of AIS may need to be made more carefully.

Owing to its extremely fast scanning speed and high-density resolution, head CT has emerged as a popular imaging method for excluding brain lesions in patients with mild symptoms [4]. AIS typically appears as a slightly hypodense area with a unilateral distribution on head CT scans. However, in the clinical setting, old cerebral infarction (OCI) and asymmetric leukoaraiosis (LA) similarly display slightly hypodense lesions. Thus, it is sometimes challenging to distinguish AIS from these two types of lesions [5]. AIS and OCI can occur anywhere in the brain and present as solitary or multiple lesions, especially in the basal ganglia region. In AIS, toxic edema caused by brain cell ischemia and hypoxia can decrease parenchymal density, resulting in swelling cerebral edema and shallow brain sulci. OCI presents as softening areas with cerebrospinal fluid density after AIS. LA is typically identified adjacent to the lateral ventricles and is always symmetrically distributed in both cerebral hemispheres. However, atypical LA may present as asymmetric hypodense lesions. OCI that has not been fully softened and asymmetrically distributed LA should be distinguished from AIS. Making a definitive diagnosis requires further examinations, such as brain MRI [6]. However, this process can delay the diagnosis and treatment of AIS.

Recognizing an indeterminate hypodense lesion as AIS based on CT scans could accelerate the diagnostic process and prevent delays in treatment. Indeed, it is particularly helpful in patients with MRI contraindications. As a critical semiquantitative parameter of CT, the CT attenuation value reflects the density of local tissues. Previous studies noted that fluctuations in cerebral hemoglobin concentration and cerebral blood volume can lead to changes in CT attenuation values [7]. Determining CT attenuation values in specific affected areas can assist in the assessment of the Alberta Stroke Program Early CT Score of AIS [8]. We hypothesized that the density of the aforementioned hypodense lesions differs due to inconsistencies in the proportion of normal brain cells and water contained in the lesions, which can be reflected by CT attenuation values. Therefore, this study aims to determine whether CT attenuation values can distinguish between AIS, OCI, and LA.

## Materials and methods

### Dataset

This study was approved by the Medical Research Ethics Committee, and the requirements for informed consent were waived. The preliminary results of this study were presented at the European Congress of Radiology 2023 in the form of a scientific poster [9]. A retrospective analysis was performed on patients with hypodense lesions detected by head CT and confirmed by MRI/DWI at the Second Affiliated Hospital of Nanchang University between June 2019 and June 2021.

During case collection, patients who underwent head CT with indeterminate hypodense lesions in the CT report by radiologists from the medical record system were identified. Then, patients underwent MRI/DWI within 48 h after CT scanning were screened. The inclusion criteria for the target 3 types of lesions were based on imaging findings and clinical diagnosis. (1) AIS, rapidly progressive neurological deficit of vascular origin lasting within 3 days of onset, was diagnosed by hyperintense on DWI [3, 10]. To evaluate the influence of the timing of symptom onset on CT attenuation values, AIS was divided into hyperacute (<6 h) and acute stages (6 h ~ 72 h) based on the time interval between the CT scan and symptom occurrence. (2) OCI was characterized by a hypointense on DWI and T1WI, a hyperintense on T2WI, and a hypointense and surrounding hyperintense on T2FLAIR [11, 12]. (3) LA was defined as isointense on DWI, slightly hypointense on T1WI, marginally hyperintense on T2WI, and hyperintense on T2FLAIR [13]. Only LA lesions with unilateral and asymmetrical distributions were included.

The exclusion criteria were as follows: (1) lesions that were too small to accurately measure the CT attenuation value, or lesions with a large area that could easily diagnosed as AIS or OCI according to the radiologist’s report; (2) the presence of lesions in the contralateral symmetrical area; and (3) poor image quality; (4) the presence of other lesions such as tumors. The age and gender of all patients were recorded.

### CT attenuation values measurement and parameters calculation

CT attenuation values were measured on the Vue Image archiving and communication system (Koninklijke Philips, Version 12). Multi-plane reconstruction was utilized to adjust the thin slice CT image with a layer thickness of 1.25 mm to the standard axial position. The normal brain parenchyma in the symmetrical region of the contralateral cerebral hemisphere of each target leision was included in the control group.

With reference to MRI images, the slice with the largest cross-section of hypodense lesion was selected. The circular region of interest (ROI) was placed on the lesion as well as the control region to determine the CT attenuation values. Measurements were re-conducted on the two adjacent slices (upper and lower) again. Sulci, fissures, pools, blood vessels, and calcifications were avoided during outlining. All CT attenuation values were measured by a radiologist with 3 years of experience. The mean CT attenuation values were used for further analysis.

The CT attenuation value difference (ΔHU) between the control tissue and the hypodense lesion was calculated using the following formula: ΔHU = HU_control_ - HU_lesion_. The CT attenuation value Ratio (Ratio_HU_) between the control tissue and the hypodense lesion was calculated using the formula: Ratio_HU_ = HU_lesion_/HU_control_.

### Statistical analysis

Statistical analysis were carried out using SPSS (version 18.0, SPSS Inc.) and Prism software (version 7.04, GraphPad Prism). *P* < 0.05 was considered statistically significant. The chi-square test was used to compare differences in gender, while one-way analysis of variance (ANOVA) was used to compare the age among the groups.

The paired Student’s t-test was used to compare the CT attenuation value between the hypodense lesion groups and their control groups. ANOVA was used to compare the CT attenuation value, ΔHU and Ratio_HU_ among the 3 lesion groups, and pairwise comparisons were made by the least significant difference (LSD) method. ΔHU and Ratio_HU_ between the hyperacute and acute stages of AIS were compared using an independent sample t-test.

Receiver operating characteristic (ROC) curves were plotted to analyze the efficacy of CT parameters (attenuation values, ΔHU and Ratio_HU_) in identifying hypodense lesions and control tissues. Their optimal cutoff values for diagnosis were determined according to the maximum Youden’s index (Youden’s index = sensitivity% + specificity% – 100).

## Results

### Demographic characteristics

**Fig. 1:**
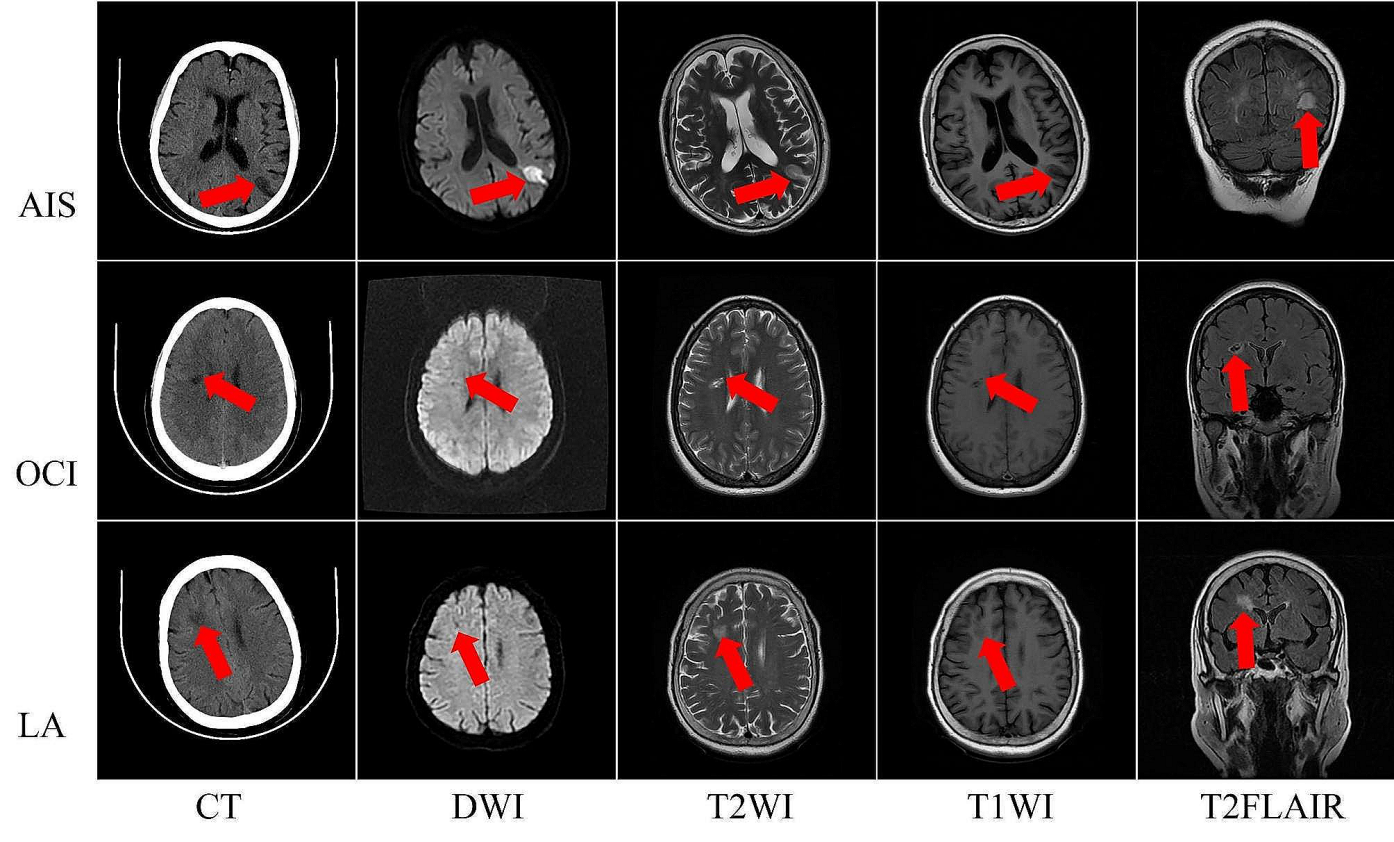
Schematic representation of different types of cerebral hypodense lesions. From top to bottom, the images illustrate patients with acute ischemic stroke (AIS), old cerebral infarction (OCI), and leukoaraiosis (LA), respectively. The CT attenuation values of the lesions were 14 HU, 10 HU, and 19 HU, respectively. The images from left to right display CT, DWI, T2WI, T1WI, and T2FLAIR sequences

From the hospital records, 246 patients with indeterminate hypodense brain lesions that performed both CT and MRI/DWI within 48 h were identified. Among them, 84 cases were excluded because the lesions were either too small or too large, 11 cases were excluded because there were lesions,  2 cases were excluded for poor image quality and 3 cases were excluded for the co-existence of tumors. Finally,167 lesions in 146 patients were included in the study. Among them, 126 patients had only 1 of the 3 types of lesions, 11 patients had 1 AIS and 1 OCI lesions, 5 patients had 1 AIS and 1 LA lesions, 3 patients had 1 OCI and 1 LA lesions, and 1 patient had 1 AIS and 2 OCI lesions. A total of 39 AIS lesions (age 66.67 ± 12.24, 61.5% male), 53 LA lesions (age 69.02 ± 10.50, 47.2% male), and 75 OCI lesions (age 64.43 ± 14.52, 72% male) were included for analysis. There was a significant difference in gender (*P* = 0.017) among the three groups but no difference in age (*P* = 0.139). The time interval between symptom onset and CT scans for AIS group was 32.21 ± 26.85 h. The time interval of hyperacute IS (*n* = 6) and acute IS (*n* = 33) was 2.92 ± 1.43 h and 37.53 ± 25.80 h, respectively. Figure 1 illustrates a schematic diagram of hypodense lesions.

### CT parameters

**Fig. 2:**
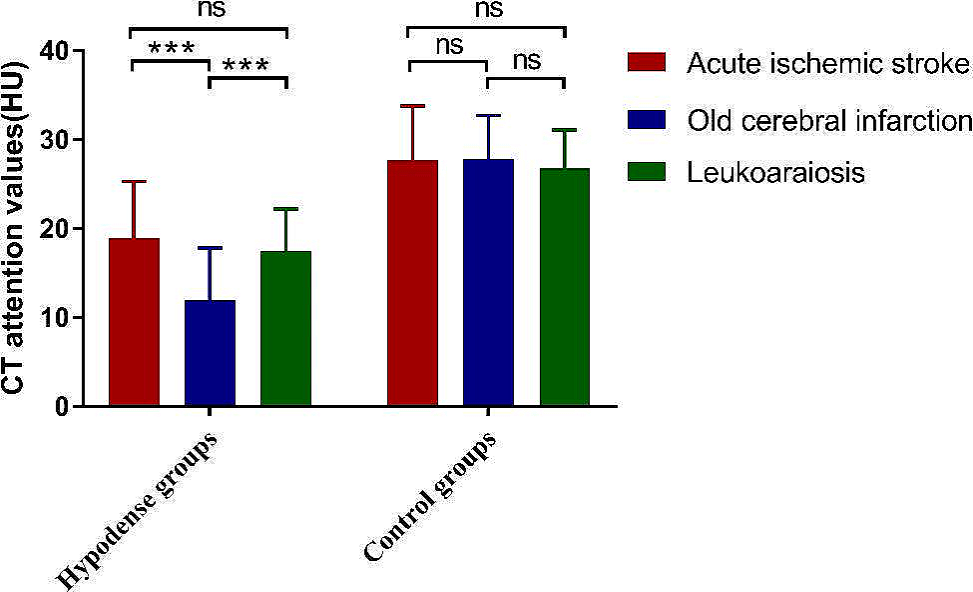
CT attenuation values in hypodense lesion groups and their corresponding control groups. There was no difference in the CT attenuation values of the control groups for the three types of lesions. “***” represents a *P* value < 0.001, and “ns” represents no significant difference. HU: Hounsfield Unit

Table 1 presents the CT attenuation values, ΔHU and Ratio_HU_ in groups. As anticipated, significant differences were identified between the lesion and control groups (all *P* < 0.001). ANOVA revealed significant differences in the CT attenuation values, ΔHU and Ratio_HU_ among the 3 lesion groups (all *P* < 0.001). In addition, LSD comparison indicated that CT attenuation values of LA and AIS (*P* = 0.254), as well as the ΔHU (*P* = 0.587) and Ratio_HU_ (*P* = 0.450), were comparable. No significant difference was found in the CT attenuation values among the control groups (*P* = 0.139). Figure 2 displays the CT attenuation values of the hypodense lesion groups and their corresponding control groups.

**Table 1:** CT parameters of the hypodense lesion and control groups

	CT attenuation values of lesions (HU)	CT attenuation values of control groups (HU)	ΔHU (HU)	Ratio_HU_
AIS	18.90 ± 6.40	27.64 ± 6.16	8.74 ± 3.00	0.67 ± 0.13
LA	17.53 ± 4.67	26.74 ± 4.33	9.21 ± 2.69	0.64 ± 0.11
OCI	11.90 ± 5.92	27.80 ± 4.93	15.90 ± 5.24	0.42 ± 0.19
*P* value	< 0.001	0.139	< 0.001	< 0.001

*AIS: acute ischemic stroke; OCI: old cerebral infarction; LA: leukoaraiosis; HU: Hounsfield unit; ΔHU: CT attenuation values difference; Ratio_HU_:CT attenuation value ratio

Notably, significant differences were detected between the CT attenuation values of hyperacute IS and acute IS, with values of 23.50 ± 9.67 HU and 18.06 ± 9.41 HU, respectively (*P* = 0.042). In contrast, no significant differences in ΔHU and Ratio_HU_ were noted between the two subgroups, with values of 7.94 ± 2.14 HU and 0.73 ± 0.07 for hyperacute IS and, 8.88 ± 3.13 HU and 0.66 ± 0.133 for acute IS (*P* = 0.512, *P* = 0.213).

### ROC analysis

**Fig. 3:**
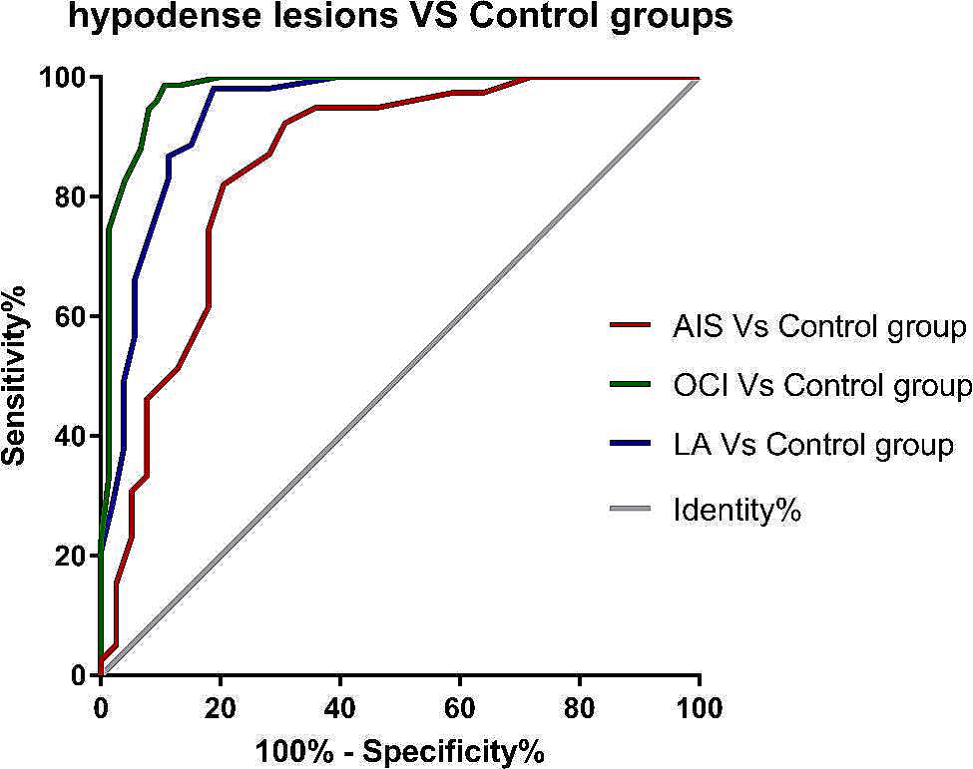
ROC curves for CT attenuation values in distinguishing hypodense disease from normal tissue

The areas under the ROC curves (AUCs) of CT attenuation values for discriminating AIS, OCI, and LA from symmetrical control normal tissue were 0.853, 0.978, and 0.940, respectively (all *P* < 0.001) (Fig. 3).

**Fig. 4:**
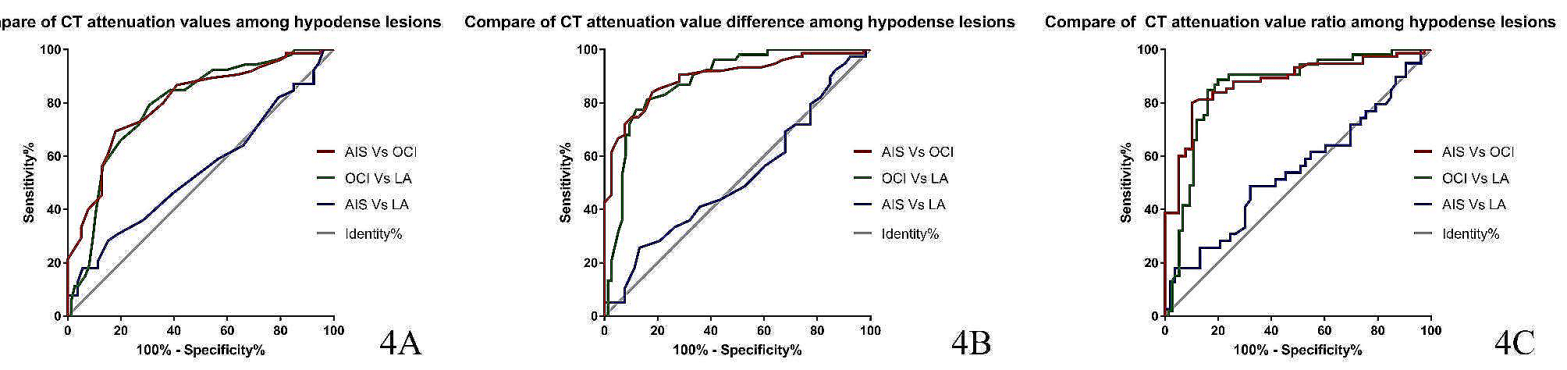
ROC curves for CT attenuation values (**4a**), attenuation value difference (**4b**) and attenuation value ratio (**4c**) in distinguishing hypodense lesions. All parameters fail to distinguish AIS from LA. * AIS: acute ischemic stroke; OCI: old cerebral infarction; LA: leukoaraiosis

The ROC curves of CT parameters for discriminating the three types of hypodense lesions are presented in Fig. 4, Tables 2, 3 and Table 4. The AUCs for CT value, ΔHU and Ratio_HU_ in distinguishing AIS from OCI were 0.802, 0.896, and 0.878 (all *P* < 0.001). Meanwhile, the AUCs for CT value, ΔHU, and Ratio_HU_ in distinguishing OCI from LA was 0.789, 0.883, and 0.857, respectively (all *P* < 0.001). Among the three parameters, while ΔHU displayed the highest performance in the identification process, it could not distinguish AIS from LA.

**Table 2:** Optimal cutoff of CT attenuation values for the identification of hypodense lesions

	AUC	sensitivity	specificity	Optimal cutoff values (HU)	*P* value	Youden index
AIS Vs OCI	0.802	82.05%	69.33%	14.00	<0.001	0.5138
OCI Vs LA	0.789	69.33%	79.25%	14.00	<0.001	0.4858
AIS Vs LA	0.545	-	-	21.00	0.46^#^	0.1311

*AIS: acute ischemic stroke; OCI: old cerebral infarction; LA: leukoaraiosis; AUC: area under the ROC curve; HU: Hounsfield Unit

^#^*P* > 0.05 suggested that the validity of the parameters in differentiating the lesions could not be confirmed

**Table 3:** Optimal cutoff of CT attenuation value difference for the identification of hypodense lesions

	AUC	sensitivity	specificity	Optimal cutoff values (HU)	*P* value	Youden index
AIS Vs OCI	0.896	84.00%	82.05%	11.00	<0.001	0.6605
OCI Vs LA	0.883	88.00%	77.36%	10.33	<0.001	0.6536
AIS Vs LA	0.519	-	-	6.33	0.755^#^	0.1243

*AIS: acute ischemic stroke; OCI: old cerebral infarction; LA: leukoaraiosis; AUC: area under the ROC curve; HU: Hounsfield unit

^#^*P* > 0.05 suggested that the validity of the parameters in differentiating the lesions could not be confirmed

**Table 4:** Optimal cutoff of CT attenuation value ratio for the identification of hypodense lesions

	AUC	sensitivity	specificity	Optimal cutoff values	*P* value	Youden index
AIS Vs OCI	0.878	89.70%	80.00%	0.5542	<0.001	0.6974
OCI Vs LA	0.857	84.00%	84.90%	0.5783	<0.001	0.6891
AIS Vs LA	0.545	-	-	0.71	0.468^#^	0.1664

*AIS: acute ischemic stroke; OCI: old cerebral infarction; LA: leukoaraiosis; AUC: area under the ROC curve

^#^*P* > 0.05 suggested that the validity of the parameters in differentiating the lesions could not be confirmed

## Discussion

Herein, the CT values of three types of hypodense lesions, namely AIS, asymmetric LA, and OCI, which are challenging to distinguish using the naked eye, were quantitatively analyzed. On the one hand, significant differences were identified in CT attenuation values, ΔHU and Ratio_HU_ between OCI and AIS/LA. On the other hand, no significant differences were observed in these parameters between LA and AIS. ROC analysis identified that the optimal cutoff value for CT attenuation values to differentiate OCI from AIS and LA was 14 HU.

In the present study, the mean CT attenuation value of AIS was 18.90 HU. Cerebral infarction may lead to edema and necrosis, and brain cells are less dense than contralateral normal tissues. Therefore, the decrease in CT attenuation value was consistent with the results of a previous study [9]. Srivatsan et al. described that AIS can be visualized on a relative non-contrast CT map, thereby reflecting the difference in CT attenuation values between normal tissue and the infarcted area. However, they did not explore CT attenuation values in other hypodense lesions [14]. To evaluate the influence of the timing of symptom onset on CT attenuation values, patients with AIS were categorized into hyperacute and acute stages. Of note, these values in the acute stage compared to those in the hyperacute stage, in line with clinical observations suggesting that prolonged onset time may promote the development of edema and lead to a more significant decrease in density. Notwithstanding, the number of hyperacute phases in our study was limited (only 6 cases), and the possibility of errors cannot be excluded.

OCI is a nonacute lesion appearing 3 weeks after the onset of AIS, hallmarked by areas of liquefaction and softening, and presenting as fluid density on CT [12]. The OCI lesions included in this study had a CT attenuation value of approximately 11.90 HU, which did not reduce to the density of water and thus could not be clearly differentiated from AIS based exclusively on visual inspection. However, the determination of CT attenuation values in the study provided valuable insights for quantitative identification. The cutoff value between OCI and both AIS and LA was 14 HU, suggesting that < 14 HU can be used as a diagnostic criterion for OCI.

LA is currently recognized as a cerebrovascular disease [15] associated with cognitive decline, gait, urinary system disorders, and Parkinson’s disease [16, 17]. It usually presents with multiple lesions symmetrically distributed in the paraventricular and deep cerebral white matter, with a small proportion presenting asymmetrically [18]. It appears as hypointense lesions on CT and demonstrates hyperintense on T2-FLAIR [19]. Our study signaled that CT attenuation values could not differentiate between AIS and LA. This could be ascribed to changes in CT values caused by the increase in water content in specific brain parenchymal regions.

According to a previous study, the effectiveness of ΔHU and Ratio_HU_ between vertebral fractures and control vertebrae in differentiating fresh and old vertebral fractures was superior to that of CT values [20]. Interestingly, ΔHU and Ratio_HU_ outperformed simple CT attenuation values in distinguishing AIS from LA. However, they could not distinguish AIS from LA. While ΔHU is more sensitive to detecting alterations in brain tissue density, the similarity in the degree of density reduction of AIS and LA poses challenges in distinguishing them solely by attenuation values and ΔHU. In order to timely diagnose AIS and LA using CT, the combination of additional techniques, such as dual-energy CT or radiomics, may be necessary.

### Limitations

This study has several limitations that cannot be overlooked. **To begin**, all the enrolled cases underwent MRI after CT, implying that the radiologists could not make a definitive diagnosis based on CT alone, which might introduced bias in patient selection. **Secondly**, the diagnoses of AIS, OCI, and LA were all exclusively based on imaging and clinical diagnosis. **Thirdly**, our investigation uncovered that while CT values could distinguish OCI from AIS, they could not distinguish AIS from LA. Consequently, our results did not meet our initial assumptions. This unexpected result highlights the need for further research into the timely diagnosis of AIS and LA. **Fourthly**, the time interval between symptom onset and CT markedly influences lesion characteristics. Given the variability of AIS lesions over time, the distinction between hyperacute and acute phases lacks specificity. Furthermore, the current study did not explore relationship between stroke onset and CT parameters. Lastly, given the limited number of hyperacute IS cases, further large-scale studies are warranted to validate our results.

## Conclusion

CT parameters including attenuation values, ΔHU, and Ratio_HU_ could effectively distinguish OCI from AIS and OCI from LA in patients with indeterminate hypodense lesions. These findings may avoid the need for unnecessary MRI examinations in patients with OCI. Notwithstanding, it remains challenging to distinguish between AIS and LA.

## Data Availability

The datasets generated and/or analysed during the current study are not publicly available due Data security, but are available from the corresponding author on reasonable request.

## Abbreviations

AIS: acute ischemic stroke
OCI: old cerebral infarction
LA: leukoaraiosis
DWI: diffusion-weighted imaging
ΔHU: CT attenuation value difference
Ratio_HU_: CT attenuation value ratio
ANOVA: one-way analysis of variance
ROC: receiver operating characteristic
AUC: area under the ROC curve
HU: Hounsfield Unit
